# The biology and toxinology of blunt-nosed vipers

**DOI:** 10.1038/s44185-025-00090-w

**Published:** 2025-06-03

**Authors:** Ignazio Avella, Maik Damm, Matteo R. Di Nicola, Josephine Dresler, Naşit İğci, Mert Kariş, Seyed Mahdi Kazemi, Benno Kreuels, Giovanni Paolino, Yiannis Sarigiannis, Andreas Vilcinskas, Wolfgang Wüster, Tim Lüddecke

**Affiliations:** 1 Animal Venomics Lab, Fraunhofer Institute for Molecular Biology and Applied Ecology (IME), Ohlebergsweg 12, 35392 Giessen, Germany; 2 Institute for Insect Biotechnology, Justus Liebig University of Giessen, Heinrich-Buff Ring 26-32, 35392 Giessen, Germany; 3 LOEWE Centre for Translational Biodiversity Genomics, Senckenberganlage 25, 60325 Frankfurt am Main, Germany; 4 Istituto Zooprofilattico Sperimentale del Piemonte, Liguria e Valle d’Aosta, Via Bologna 148, 10154 Turin, Italy; 5 Department of Pathobiology, Pharmacology and Zoological Medicine, Faculty of Veterinary Medicine, Wildlife Health Ghent, Ghent University, 9820 Merelbeke, Belgium; 6 Department of Bioresources, Fraunhofer Institute for Molecular Biology and Applied Ecology, Ohlebergsweg 12, 35392 Giessen, Germany; 7 Department of Molecular Biology and Genetics, Faculty of Arts and Sciences, Nevşehir Hacı Bektaş Veli University, 50300 Nevşehir, Türkiye; 8 Program of Laboratory Technology, Department of Chemistry and Chemical Process Technologies, Acıgöl Vocational School of Technical Sciences, Nevşehir Hacı Bektaş Veli University, 50140 Nevşehir, Türkiye; 9 Zagros Herpetological Institute, 37156-88415, P. O. No 12, Somayyeh 14 Avenue, Qom, Iran; 10 Department of Medicine, Division of Tropical Medicine, University Medical Center Hamburg-Eppendorf, 20251 Hamburg, Germany; 11 Department for Implementation Research, Bernhard Nocht Institute for Tropical Medicine, 20359 Hamburg, Germany; 12 Unit of Dermatology and Cosmetology, IRCCS Ospedale San Raffaele, Via Olgettina 60, 20132 Milan, Italy; 13 Department of Health Sciences, School of Life and Health Sciences, University of Nicosia, 2417 Nicosia, Cyprus; 14 Molecular Ecology and Evolution at Bangor, School of Environmental and Natural Sciences, Bangor University, Bangor, LL57 2UW United Kingdom

**Keywords:** Biochemistry, Biodiversity, Evolution, Phylogenetics, Taxonomy, Proteomics, Herpetology, Public health

## Abstract

Blunt-nosed vipers (genus *Macrovipera*) are among the venomous snakes of highest medical relevance in the Palearctic region. Extensive research has been conducted on their venoms, covering toxin composition, biochemistry, function, pathology and biodiscovery. However, these studies are widely dispersed across the scientific literature, almost exclusively focus on biochemistry and drug discovery aspects, and largely neglect the zoological and systematic context of these snakes. Here, we provide a comprehensive, transdisciplinary compilation of what is known about the biology, taxonomy and toxinology of blunt-nosed vipers. After contextualising the three generally recognised *Macrovipera* species (*Macrovipera lebetina*, *Macrovipera razii* and *Macrovipera schweizeri*) within their zoological and taxonomic framework, we compile the venom proteomes available in the literature and identify general compositional patterns across the genus. We then report on the known biological activities of *Macrovipera* venoms and discuss their clinical and pharmacological potential. Furthermore, we detail the mainly haemorrhagic, coagulopathic and cytotoxic pathophysiological effects of blunt-nosed viper envenoming, and provide recommendations for the clinical management of *Macrovipera* bites. Finally, we propose future research directions, advocating for expanded research on these venoms to enhance our understanding and drive further innovation in both therapeutic applications and the treatment of bites inflicted by these remarkable snakes.

## Introduction

Snake venoms are fascinating evolutionary innovations found among members of the superfamily Colubroidea, predominantly within the suborder Caenophidia, commonly known as 'advanced snakes'. These complex, protein-rich mixtures contain up to hundreds of bioactive compounds suspended in a viscous medium^1–3^. They possess distinct biochemical features that make them able to disrupt the physiological balance of target organisms^4,5^. In line with the mainly predatory purpose of snake venoms, evidence suggests that selection for effective prey subjugation is a driving force in shaping their compositions^6–8^. As a result of the influence of selective pressures on the deployment of specific toxins, as well as the evolutionary histories of divergent lineages, variation in the composition of snake venoms is extremely common and occurs at all taxonomic levels^9–11^.

Although snake venom is primarily considered a trophic adaptation, it can also serve as a powerful defence^12^; however, the extent to which this function has affected the evolution of venom composition is unclear^13^. Arguably, the most striking example of snake venom being used defensively is snakebite envenoming in humans. Officially listed as a neglected tropical disease by the World Health Organization (WHO), snakebite is estimated to affect ~5 million people annually, resulting in around 140,000 deaths and 400,000 cases of long-term disabilities worldwide^14–16^. This dramatic, yet still underestimated global health burden predominantly impacts the rural communities of Sub-Saharan Africa, the Indian subcontinent, South-East Asia, Papua New Guinea and Latin America^14,17,18^. In many of these regions, snakes of the family Viperidae are responsible for a remarkably high number of envenomation cases^19,20^. Particularly, pit vipers (subfamily Crotalinae) are the main culprits of snakebite accidents in the American continent^21^, whereas “true vipers” (subfamily Viperinae) are among the snakes of highest medical importance in the Old World^22–24^.

**Fig. 1:**
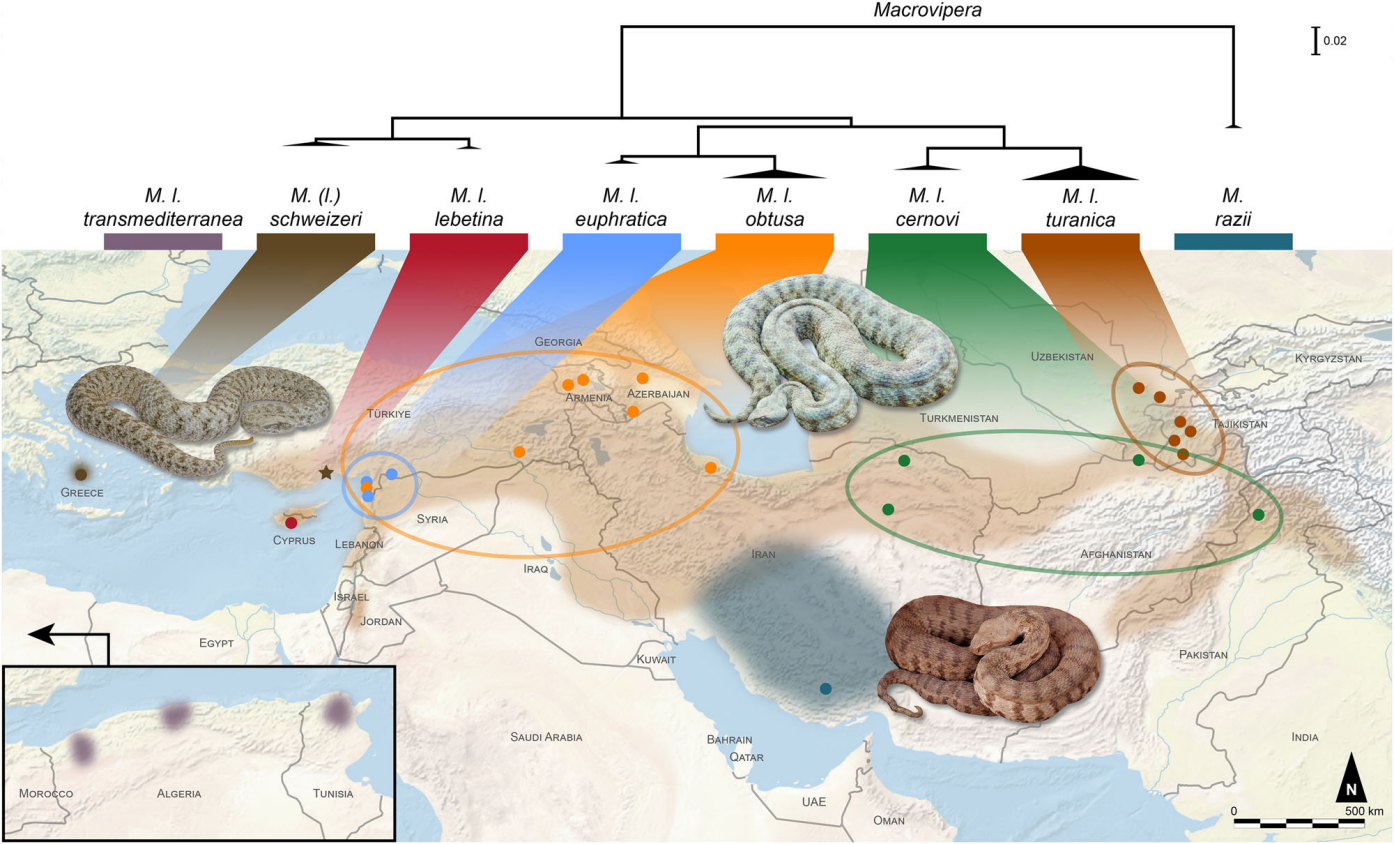
Phylogeny and phylogeography of the genus *Macrovipera*. The distribution ranges of the species *M. lebetina* (light brown), *M. schweizeri* (dark brown) and *M. razii* (dark blue), as well as the subspecies *M. l. transmediterranea* (of debated validity and possibly extinct), were sourced from the WHO Snakebite Information and Data Platform^30^. The distribution ranges of the other *M. lebetina* subspecies (ellipses) and the phylogenetic tree were adapted from Stümpel^44^, with the triangles at the branch tips representing the age and sampling of each taxon. The points on the map indicate the sampling localities from Stümpel’s work. The star represents *M. lebetina* populations from coastal southern Türkiye sharing mitochondrial haplotypes with *M. schweizeri*. Photo credits: Rami Khasab (*M. l. obtusa*), Seyed Mahdi Kazemi (*M. razii*), Ignazio Avella (*M. schweizeri*).

Within the subfamily Viperinae, the genus *Macrovipera* stands out as one of the groups of higher medical relevance. Also known as blunt-nosed vipers, members of this genus are large, thickset venomous snakes that often exceed 100 cm in total length^25^. Despite ongoing debates among taxonomists regarding the systematic relationships within *Macrovipera*^26,27^, three distinct species of blunt-nosed vipers are usually recognised: the Levantine viper, *Macrovipera lebetina*; Razi’s viper, *Macrovipera razii*; and the Milos viper, *Macrovipera schweizeri*. Blunt-nosed vipers are widely distributed across the Palaearctic, from southern Türkiye to north-western India (Jammu and Kashmir), and their presence has been suggested for Algeria and Tunisia^28,29^. They are also found in Cyprus and on the Greek islands of Milos, Sifnos, Kimolos, and Polýaigos in the south-western Cyclades^30^ (Fig. 1).

Given their wide distribution and the potency of their venoms, blunt-nosed vipers are responsible for a high number of severe snakebite incidents in the Near and Middle East^23,31,32^, and also pose a considerable medical threat in Cyprus^33^. Their high medical relevance has led to extensive studies on their toxin arsenals, resulting in a wealth of knowledge spanning across different research fields. Notably, numerous studies have investigated the biochemical and pharmacological properties of *Macrovipera* venoms^34–36^, and several components have been found to hold great promise for drug research and the development of new therapeutic applications^37–40^. Recently, a number of reviews on blunt-nosed viper venom have been published, primarily focusing on biochemistry and drug discovery aspects^40,41^. However, considering the relevance of snakebite envenoming and the limited understanding of the ecology of these snakes, a more holistic, transdisciplinary perspective is required.

Here, we present a comprehensive compilation of the information available on the snakes of the genus *Macrovipera* and their venoms. Starting with an overview of their zoological, taxonomic and systematic context, we describe the composition and biological activities of their venoms, along with the pathophysiological effects they induce. Furthermore, we explore their pharmacological potential, highlighting prospects for translational research and focusing on promising components for drug development. Finally, drawing on published epidemiological data and case reports, we underscore their medical importance and discuss clinical management, including antivenom therapy. This review consolidates the extensive body of research on *Macrovipera* venoms, providing an updated zoological and taxonomical context. It aims to highlight both the challenges and opportunities these venoms present, with the hope of inspiring ongoing advancements in treatment and further exploration of the toxin arsenals of these formidable vipers.

## Zoological, phylogenetic and taxonomic framework

Despite its medical importance^30^, *Macrovipera* has received limited taxonomic attention compared to other Eurasian vipers, and previous works have failed to clarify the phylogeny of this genus^42,43^. The most comprehensive molecular study to date is a purely mitochondrial phylogeographic analysis presented in the PhD thesis of Stümpel^44^. A graphical summary of the findings from this unpublished work is provided in Fig. 1. However, given the inadequacy of mitochondrial DNA alone for species delimitation^45^, resolving species limits and taxon boundaries within the genus will require geographically dense sampling and the use of nuclear genetic markers to determine the extent to which the different mitochondrial haplogroups correspond to independent lineages on separate evolutionary trajectories^46,47^. The summary of the current state of knowledge we provide here is therefore incomplete, and as much as anything highlights the numerous remaining knowledge gaps surrounding this genus.

**Fig. 2:**
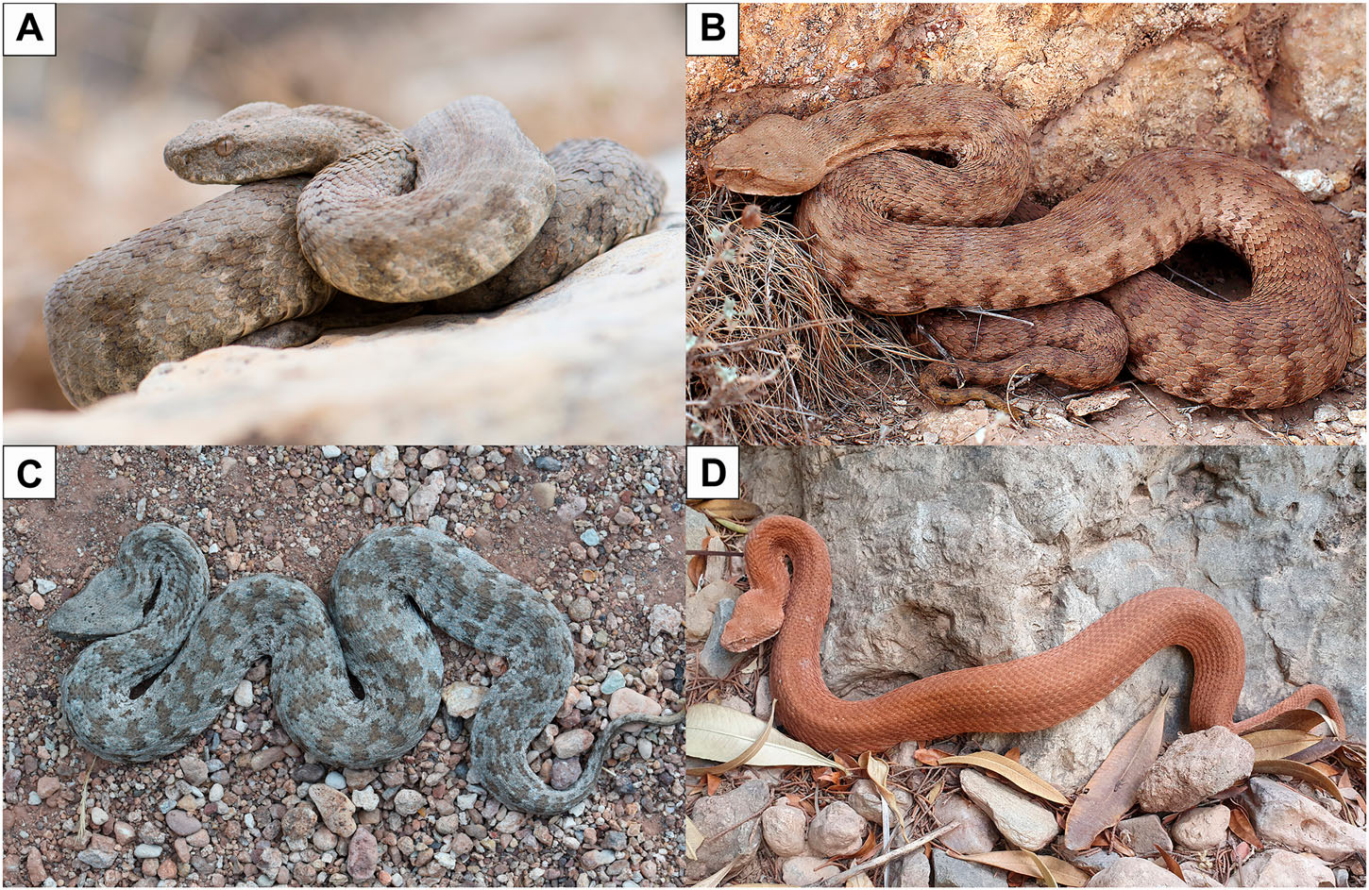
The three extant species of *Macrovipera.* The figure shows representative individuals and phenotypes for each currently recognised species: **A** *M. l. lebetina*; **B** *M. razii*; **C** *M. schweizeri*, cryptic phenotype; **D** *M. schweizeri*, red phenotype. Photo credits: Thor Håkonsen (**A**), Seyed Mahdi Kazemi (**B**), Matteo R. Di Nicola (**C**, **D**).

Stümpel^44^ found evidence of a basal, highly divergent lineage of *Macrovipera* from the Zagros Mountains in southern Iran, recently described as the new species *M. razii*^48^. Within the remainder of the genus, the considerable mitochondrial phylogeographic structure includes a basal East-West divide, separating western populations in the Cyclades, the southern coast of Anatolia, and Cyprus from the rest of the range. Divergence within this Western group is very minor, suggesting a common ancestry in relatively recent evolutionary times. Furthermore, Stümpel^44^ identified a broad area encompassing present-day eastern Türkiye, north-western Iran and the southern Caucasus as the centre of origin of *M. lebetina*. In line with this, recent reconstructions of the Viperinae diversification timeline applying the fossilised birth-death approach also indicate an Asian origin for *Macrovipera*^49^. Intriguingly, while the earliest fossil record of *Macrovipera* dates back to the middle Miocene (~12 Mya)^50^, the differentiation of the three modern species is estimated to have occurred approximately 7 Mya. Most of the recent lineage diversity of *Macrovipera* can be traced to the Pliocene (~5.3 to 2.6 Mya) and the Pleistocene (around 2.6 Mya to ~11,700 years ago)^44^, with the genus’ species composition appearing to have remained largely unchanged^49^. Possibly concordant with this relatively recent diversification, the three generally accepted species exhibit a similar phenotype, characterised by a robust body, a typically dull, greyish-brown background colouration with a darker, barred pattern, and a prominent, triangular-shaped head (Fig. 2). Nonetheless, notable differences can be detected among the three taxa, particularly concerning their ecology and feeding habits. Here we provide a zoological overview of the currently recognised *Macrovipera* taxa, with a focus on their distributions and main ecological and morphological features.

### Levantine viper—*Macrovipera lebetina*

The Levantine viper, *M. lebetina*, is the most widespread species of genus *Macrovipera*. Its distribution ranges from Cyprus to north-western India, passing through Türkiye, Iraq, Iran, Turkmenistan and Afghanistan. It reaches its highest and lowest latitudes in Russia (Dagestan) and Pakistan, respectively (Fig. 1). In the past, diverse constellations of subspecies have been recognised; currently, four subspecies are widely accepted: the nominal subspecies *M. l. lebetina* (Fig. 2A), considered endemic to Cyprus; *M. l. obtusa*, from across a wide area from southern Türkiye to western Iran, Armenia, Azerbaijan and Dagestan, including also Iraq, Jordan, Syria and Lebanon; *M. l. cernovi*, from north-eastern Iran, southern Turkmenistan, parts of Afghanistan, Uzbekistan, Pakistan and India (Jammu and Kashmir); and *M. l. turanica*, restricted to the north-eastern part of the distribution, between eastern Turkmenistan, Uzbekistan, Tajikistan and Kazakhstan^44,51,52^. Questions remain over the affinities of the populations from the Mediterranean coast of southern Anatolia, and the status of the controversial subspecies *M. l. euphratica* and *M. l. peilei*. The poorly understood taxon *M. l. euphratica* is limited to a group of specimens from southern Türkiye which exhibit a somewhat distinct haplotype clade compared to the widespread *M. l. obtusa*^44^. The sympatry of this clade with the *M. l. obtusa* group is somewhat surprising. The subspecies *M. l. peilei* appears to have been overlooked due to the rarity of voucher specimens, with its type locality in the hard-to-access regions of southern Afghanistan (Zandra) and eastern Pakistan (Quetta). Equally enigmatic is the North African occurrence of *M. l. transmediterranea*, reported only from a very limited number of localities in the coastal mountains of Algeria and Tunisia (Fig. 1). As no live specimens have been documented since the 1980s, the validity and persistence of this taxon are currently questioned^53,54^. Nonetheless, climatic niche modelling indicates that the climate in the Maghreb and along the Mediterranean coasts of Libya and Egypt is or was (in the mid-Holocene) likely suitable for vipers of the genus *Macrovipera*^55,56^, suggesting a potential colonisation pathway for these now remote and isolated populations. Another possibility is the anthropogenic introduction of these populations from Asia in ancient times^57^. To resolve the mystery surrounding this taxon, the acquisition of fresh genetic material or the analysis of preserved *M. l. transmediterranea* type specimens will ultimately be required. Preservation in alcohol and fixation in formalin, commonly used in museum herpetological collections, can lead to DNA degradation through hydrolysis, miscoding lesions, and protein-DNA cross-links, which may render sequence information unobtainable using conventional methods^58,59^. However, ancient DNA techniques employ specialised protocols for DNA extraction, sequencing library preparation, and often target enrichment procedures, which allow for the successful retrieval of DNA sequences from heavily degraded samples^59,60^. These emerging approaches are becoming valuable tools for accessing the vast repositories of biodiversity, enabling genetic analyses, including species delimitation^61,62^, and could ultimately help clarify the status of *M. l. transmediterranea*. In this review, we have accepted previous publications on the venoms of *M. l. transmediterranea* at face value. However, it appears possible that the venoms in question may have actually originated from another subspecies of *M. lebetina*, or from misidentified specimens of the common and widespread Moorish viper, *Daboia mauritanica*^36^.

The Levantine viper is the largest species in the genus *Macrovipera*, with individuals typically reaching 70–90 cm in total length^63,64^, but reported to grow up to 150 cm^65^. Although a few records suggest that mainland subspecies may exceed 200 cm (e.g., *M. l. obtusa*^66^), the accuracy of these reports is debated. This ambush predator feeds primarily on small endotherms, such as rats and mice^67^, and inhabits a variety of habitats, including river valleys, gorges, pine forests, orchards, and ruins^65,67^. These snakes are strongly associated with structures that facilitate thermoregulation and thermal protection (e.g., crevices, caves, rock piles and rock walls)^68^. Furthermore, the Levantine viper is known to show an affinity for water bodies, which serve as ambush sites, and are important for water intake and thermoregulation^63,65,69^. In remarkable contrast to the ecology and behaviour of the species in most of its range, Al-Sheikhly et al.^70^ reported that *M. lebetina* specimens from the Mesopotamian marshes of southern Iraq and south-western Iran present atypical colourations and are swift swimmers capable of extended dives to evade threats, suggesting local adaptation to wetland environments.

Despite being generally persecuted throughout its range, *M. lebetina* is classified as “Least Concern” by the International Union for Conservation of Nature (IUCN)^54,71^. However, it is suspected to be declining in Cyprus^63^, and illegal collection of individuals for the pet trade is known to occur in Türkiye^54^. Other threats include habitat conversion due to agriculture and associated irrigation schemes, urban development, mining, overgrazing, soil erosion and harvesting for venom^71^. The lack of data on the status of several subpopulations across its range highlights the need for thorough population monitoring. For instance, the apparently small subpopulation in Dagestan may warrant a national listing as “Threatened” or “Near Threatened”, and populations in Turkmenistan and Jordan are also likely to be considered threatened^54^.

### Razi’s viper—*Macrovipera razii*

Razi’s viper, *M. razii* (Fig. 2B), is the latest addition to the genus, having been described as recently as 2018^48^. Endemic to Iran, this species occurs in the central and southern parts of the country in the Zagros region, where it can be found in montane woodlands, forest steppes, desert basins, as well as desert and semi-desert areas, at elevations from 1500 m to over 3000 m asl^72,73^. Species distribution modelling based on current climatic conditions indicates that the northern and north-eastern regions of Iran possess suitable habitat conditions for *M. razii*, despite the species never having been recorded in these areas^74^. Prior to its formal description, populations of *M. razii* were assigned to *M. lebetina*, although phenotypic differences, particularly in scale counts and colour patterns, had been noted^75^. The separation of this Iranian clade from the rest of the genus is estimated to have taken place ~6.6 million years ago during the Messinian period, likely as a result of increasing aridification and the formation of desert areas^44^. Molecular analyses recovered distinct mitochondrial haplotypes in a well-structured geographic pattern, suggesting the presence of previously overlooked genetic variability within the species, particularly between populations from the central and southern Zagros region^72^.

To date, virtually no data exist on the habitat use, feeding ecology, reproduction, or behaviour of *M. razii*. While it appears reasonable to hypothesise some degree of ecological similarity between this species and Iranian *M. l. obtusa*, with which it shares part of its distribution range^48,72^ (Fig. 1), long-term field studies and radio telemetry would provide valuable, direct ecological data and insights into its ecological requirements. Furthermore, although species distribution models indicate a broader potential range for *M. razii*, no confirmed records exist from northern and north-eastern Iran. Future field surveys across the country are, therefore, essential to determine the species’ full distribution. Additionally, the presence of geographically structured genetic variability suggests possible cryptic diversity within *M. razii*. Further genetic studies using nuclear markers, and ideally genome-wide analyses, are needed to clarify the population structure and phylogeography of this species. Lastly, due to its recent discovery, the conservation status of *M. razii* has yet to be assessed by the IUCN. As it is currently unclear whether the species faces significant threats, research into habitat loss, conflict with humans, and the impacts of climate change is necessary to inform conservation strategies and potential protection measures.

### Milos viper—*Macroviperaschweizeri*

The Milos viper, *M. schweizeri* (Fig. 2C, D), as generally conceived, is endemic to the Greek islands of the Milos Archipelago (Milos, Sifnos, Kimolos and Polýaigos), in the south-western Cyclades^30,76^. Stümpel^44^ found that the mitochondrial haplotypes of the Cyclades populations are nested within those of *M. l. lebetina*, and that the Milos populations share haplotypes with populations from coastal southern Türkiye (Mersin) (Fig. 1). These findings suggest a very recent divergence between Cyclades and nearby mainland populations, likely dating to the late Pleistocene or even Holocene. As a result, the Milos viper is sometimes considered a subspecies of *M. lebetina*^26,77^, but several authors continue to consider it as a valid species^27,76^, partly due to Stümpel’s results and data remaining unpublished. In the absence of a more thorough published assessment of the status of this island form, we retain it as a separate species.

Classified as “Endangered” on the IUCN Red List, *M. schweizeri* faces threats from habitat loss, road mortality, human persecution, and illegal collection. As a distinct species, it receives dedicated conservation attention at national and international levels^78,79^. However, a taxonomic downgrade to a subspecies of *M. lebetina* could lead to a reassessment of its conservation status, potentially lowering its threat category and reducing prioritisation in legal protections, enforcement, and habitat conservation efforts. As public and governmental support for conservation often depends on the perceived uniqueness of a species^80,81^, a reclassification may reduce advocacy and funding. Given these potential consequences, any taxonomic revisions should be accompanied by a reassessment of conservation strategies to ensure continued protection despite changes in classification.

Irrespective of this taxonomic conundrum, *M. schweizeri* presents a set of morphological and ecological features that make it substantially distinct from its mainland congeners. For instance, it is the smallest member of the genus, with most adult specimens measuring 50–70 cm, and total lengths over 100 cm being exceptional^25,78,82,83^. The relationship between insular snake body size and prey availability has been widely discussed, often in the context of the so-called “island rule“^84^, which suggests that large mainland species tend to become dwarf on islands, while small mainland species become giant^85^. However, numerous exceptions exist, including several insular snake populations that have increased in size compared to their mainland relatives (e.g., *Notechis scutatus* from Chappell Island^86^; *Crotalus angelensis*^87^. These cases suggest that insular snake body size primarily reflects adaptation to available prey^88^. The smaller size of *M. schweizeri* compared to mainland *Macrovipera* taxa may be linked to the prevalence of smaller, lower-nutrition prey on the islands it inhabits (e.g., lizards, passerine birds) versus the larger mammalian prey available to mainland blunt-nosed vipers. This pattern is consistent with similar resource-driven size adaptations observed in other insular venomous snakes (e.g., *Notechis scutatus* from Roxby Island^89^; *Bothrops sazimai*^90^), though the specific factors shaping *M. schweizeri* size have yet to be formally investigated.

Most Milos vipers display the greyish-brown background colouration with darker bars typical of blunt-nosed vipers (Fig. 2C). However, some *M. schweizeri* specimens exhibit a distinctive red colouration, relatively uniform across the entire body (Fig. 2D). Additionally, while other blunt-nosed vipers are reported to prey primarily on small mammals^67^, the diet of *M. schweizeri* consists mainly of passerine birds. This is hypothesised to represent a trophic adaptation due to the absence of rodents from the Milos Archipelago until very recently and their still low densities^78,91^. While adult Milos vipers primarily feed on avian prey, juveniles seem to feed almost exclusively on lizards^91^, and the amount of mammalian prey consumed by adults may vary among the islands of the Milos archipelago^83^.

Although primarily found in open shrublands with large bushes, *M. schweizeri* inhabits a wide variety of habitats, including valley slopes, rocky outcrops, and cultivated fields^92,93^. Similar to *M. lebetina*, Milos vipers also show an affinity for water bodies, such as rivers and rock pools, where they can often be found in relatively high densities, lying in ambush for birds^76,78,91^. Interestingly, in autumn, when these water bodies usually dry up due to the summer heat, *M. schweizeri* can still be found in their proximity, and can be observed climbing small trees and lying on the lower branches, in ambush for its avian prey^76,91,92^. Although *M. lebetina* has also been reported to climb vegetation in close proximity to water bodies^70^, to date *M. schweizeri* is the only taxon within the genus *Macrovipera* known to exhibit semi-arboreal behaviour for predatory purposes.

The fact that *M. schweizeri* is nested deep within *M. lebetina*^44^ makes it an ideal case study for investigating the role of ecology as a selective factor in shaping snake venom composition. The large body size and associated natural history of mainland and Cypriot blunt-nosed vipers almost certainly represent the ancestral condition, from which the Cycladean vipers diverged; consequently, any unique derived traits of *M. schweizeri* venoms may represent adaptations to their changed natural history, potentially as a result of the different prey spectra available on the islands^6,94,95^.

### Nomenclatural note

Based on circumstantial evidence, Frétey^96^ inferred that Linnaeus’^97^ use of initial capital for the specific epithet in his description of '*Coluber Lebetinus*' denoted its intended status as a noun in apposition rather than as an adjective, even though this was never explicitly stated. As such, it would not be subject to gender agreement with the genus (International Code of Zoological Nomenclature, hereafter 'the *Code*'^98^). Frétey, therefore, argued that the specific name of the Levantine viper should be *lebetinus*, not the gender-agreed form *lebetina*, which has been used consistently for over 100 years, as *Vipera lebetina* or *Macrovipera lebetina*^99,100^.

Frétey’s findings are undoubtedly interesting from a historical perspective. However, we argue that changing long-established spellings of previously stable scientific names based on what is essentially 'nomenclatural archaeology' is antithetical to the primary aim of the *Code* stated in its Preamble, 'to promote stability and universality in the scientific names of animals'. Such changes do not enhance our understanding of the biology of the animals, complicate information retrieval and communication, are unpopular with most users of scientific names, and thereby undermine the reputation of taxonomy as a science^101,102^. Where new historical information would change long-established scientific names under the *Code*, authors should seek an Opinion from the International Commission on Zoological Nomenclature to preserve current usage. Furthermore, we urge the Commission to seek ways to limit the potential for historical discoveries to destabilise long-standing, stable scientific names. The *Code* already includes mechanisms to prevent rediscovered forgotten names from displacing long-established names (*nomina oblita* - Article 23.9). Similar mechanisms should be implemented for forgotten acts or facts (*acta* and *facta oblita*), such as misidentified type specimens or historical details such as those discussed here. This would enhance information retrieval and communication in the biodiversity sciences in the middle of a mass extinction. In protest against nomenclatural changes driven by reasons other than advancing systematic knowledge, and to stimulate discussion on ways of reducing the burden of nomenclatural history in taxonomy, we here retain the long-standing spelling *Macrovipera lebetina* for the Levantine viper.

## Venom composition

The term 'venomics' broadly defines the use of high-throughput technologies (i.e., 'omics') and biotechnological approaches to explore venoms^103–105^. The state-of-the-art approach of 'snake venomics' involves the mass spectrometry-based characterisations of venom proteins using bottom-up proteomics, combined with at least two prior decomplexation steps^106^. Since the advent of snake venomics in the early 2000s, it has become possible to provide a detailed picture of the diversity and relative abundances of toxin families present in the venoms of various taxa. Characterising snake venom composition and uncovering inter- and intra-specific variations are essential for a more accurate assessment of the clinical outcomes of envenomation and the development of more effective antivenoms^10,14^. In particular, vipers (family Viperidae) are among the snakes that have received the most attention from snake venom research^107^, likely due to their high medical relevance to human health^19,20^, as well as their near-global distribution, spanning all continents except Australia and Antarctica^52,108^, making them highly accessible and diverse subjects for venom studies.

Drawing from the wealth of available information on the venoms of the Viperidae, Damm et al.^11^ showed that the venoms of snakes from the subfamily Viperinae typically comprise four major toxin families, accounting for ~75% of their compositions: snake venom metalloproteinase (svMP), phospholipase A_2_ (PLA_2_), snake venom serine protease (svSP) and C-type lectin and C-type lectin-related protein (CTL). This venom compositional pattern appears to be followed by all 'true viper' genera investigated, including *Macrovipera* (Table 1).

**Table 1:** Overview of the *Macrovipera* venom proteomes currently available in the literature

	Taxon	*M. l. lebetina*	*M. l. cernovi*	*M. l. obtusa*				*M. l. transmediterranea*		*M. schweizeri*	*D. mauritanica*	
	**Origin**	Cyprus	Iran	Armenia	Türkiye	Türkiye	Russia	Tunisia*	Tunisia*	Greece	Morocco	Morocco
**Major toxin families**	**svMP**	30.9%	38.5%	33.8%	✓	22.4%	24.1%	67.0%	64.0%	✓	46.5%	✓
	**PLA**_**2**_	8.6%	8.9%	14.6%	✓	12.8%	13.6%	4.0%	5.0%	✓	5.5%	✓
	**svSP**	17.2%	16.0%	14.9%	✓	18.6%	24.0%	9.0%	5.5%	✓	8.3%	✓
	**CTL**	4.4%	8.2%	14.8%	✓	14.0%	9.0%	10.0%	3.2%	✓	8.1%	✓
**Secondary toxin families**	**DI**	15.5%	13.6%	11.3%	✓	15.0%	13.7%	7.0%	15.1%	✓	13.8%	✓
	**LAAO**	7.6%	7.5%	1.7%	✓	3.7%	2.1%	-	-	✓	-	-
	**CRISP**	1.8%	<1%	2.6%	✓	0.9%	1.1%	-	-	✓	-	-
	**VEGF**	<1%	<1%	-	✓	-	-	2.0%	3.3%	✓	10.8%	✓
	**KUN**	-	-	-	✓	-	-	-	3.1%	✓	2.5%	✓
**Minor toxin families**	**NGF**	2.3%	0.6%	-	✓	0.8%	-	-	-	✓	-	-
	**5N**	1.5%	1.4%	-	-	-	0.2%	-	-	✓	-	-
	**PDE**	1.4%	0.1%	-	-	0.2%	0.2%	-	-	✓	-	-
	**HYAL**	✓	-	-	✓	-	0.1%	-	-	✓	-	-
	**PLB**	0.6%	0.4%	-	-	-	-	-	-	✓	-	-
	**CYS**	✓	✓	-	-	-	-	-	-	(✓)	-	-
**Rare toxin families**	**3FTx**	-	-	-	-	-	-	-	-	✓	-	-
	**ACE**	-	✓	-	-	-	-	-	-	-	-	-
	**AChE**	-	-	-	-	-	-	-	-	✓	-	-
	**AP**	✓	✓	-	✓	-	-	-	-	✓	-	-
	**LIP**	-	-	-	-	-	-	-	-	✓	-	-
	**PLA**_**2**_**-i**	✓	-	-	-	-	-	-	-	✓	-	-
	**PPT**	-	-	-	-	-	-	-	-	✓	-	-
	**QC**	✓	-	-	-	-	-	-	-	✓	-	-
	**Serpin**	-	✓	-	-	-	-	-	-	(✓)	-	-
	**VC3**	-	-	-	-	-	-	-	-	✓	-	-
	**Vespryn**	-	-	-	-	-	-	-	-	✓	-	-
	**Other**	✓	✓	-	-	1.0%	-	-	-	-	-	-
**Peptides**	**svMP-i**	6.3%	3.5%	-	-	9.5%	4.8%	-	-	✓	-	-
	**BPP/pGpH/NP**	1.8%	1.1%	5.3%	-	✓	5.6%	0.5%	0.8%	✓	4.5%	-
	**References**	^114^	^114^	^110^	^111^	^115^	^113^	^109^	^112^	^36^	^112^	^202^
	**Proteomics workflow**	Snake venomics	Snake venomics	Snake venomics	2D SDS-PAGE Bottom-up	Snake venomics	Snake venomics	Snake venomics	Snake venomics	Shotgun	Snake venomics	Shotgun
	**(Semi-) Quantification**	RP-HPLC + Densito	RP-HPLC + Densito + MS (SpI)	RP-HPLC + Densito + MS (SpI)	N of proteins	RP-HPLC + Densito	RP-HPLC + Densito + MS (SpI)	RP-HPLC + Densito	RP-HPLC + Densito + MS (SpI)	N of proteins	RP-HPLC + Densito + MS (SpI)	No quantification

The identified toxin families are sorted following Damm et al. ^11,115^. Check marks (✓) indicate components confirmed to be present in the venom but not quantified. Hyphens (-) indicate undetected components. Components requiring further confirmation are in brackets. Asterisks (*) indicate proteomes obtained from venom milked from the same snake. Relative abundances of toxin families are rounded to one decimal place. The available *Daboia mauritanica* venom proteomes currently in the literature^112,202^ are also presented for comparison. Information on the type of proteomics analysis and quantification protocol applied for each proteome is provided.

*svMP* snake venom metalloproteinase; *PLA*_*2*_ phospholipase A_2_; *svSP* snake venom serine protease; *CTL* C-type lectin and C-type lectin-related protein; *DI* disintegrin; *LAAO* l-amino acid oxidase; *CRISP* cysteine-rich secretory protein; *VEGF* vascular endothelial growth factor; *KUN* Kunitz-type inhibitor; *NGF* nerve growth factor; *5N* 5'-nucleotidase; *PDE* phosphodiesterase; *HYA*L hyaluronidase; *PLB* phospholipase B; *CYS* cystatin; *3FTx* three-finger toxin; *ACE* angiotensin-converting enzyme; *AChE* acetylcholinesterase; *AP* aminopeptidase; *LIP* lipase; *PLA*_*2*_*-i* PLA_2_ inhibitor; *PPT* palmitoyl-protein thioesterase; *QC* glutaminyl cyclotransferase; *VC3* venom complement activating/C3 homologue; *svMP-i* svMP inhibitor; *BPP* bradykinin-potentiating peptide; *pGpH* proline-glycine-proline-histidine; *NP* natriuretic peptide; *Densito* 1D SDS-PAGE (sodium dodecyl-sulfate polyacrylamide gel electrophoresis) densitometry; *RP-HPLC* reversed-phase high-performance liquid chromatography; *SpI* spectral intensity (MS1).

To date, nine compositions of *Macrovipera* venoms have been investigated by means of proteomics. Eight of these focus on four different *M. lebetina* subspecies: *M. l. lebetina*, *M. l cernovi*, *M. l. obtusa* and *M. l. transmediterranea*^109–115^. Only one venom proteome is available for *M. schweizeri*^36^ and none for *M. razii*. Given that no studies on *M. razii* venom currently exist, we hereby present a qualitative and quantitative comparative overview of the venom proteomes of *M. lebetina* subspecies and *M. schweizeri* reported in the literature. For detailed information on the individual venom components, we refer to the comprehensive catalogue of proteins and peptides described from the venom of *M. lebetina* subspecies compiled by Siigur and colleagues^41^.

From a qualitative perspective, the *Macrovipera* venom proteomes available to date are remarkably similar. Indeed, all analysed taxa share all major toxin families and most of the secondary ones (Table 1). Intriguingly, the venom of *M. schweizeri* appears to be the most complex in terms of number of components. However, it should be noted that the venom proteome for this species was the only one obtained through shotgun proteomics, while most *Macrovipera* venom compositions were analysed using a snake venomics approach^11,115^. Therefore, the observed discrepancies in venom compositional diversity between *M. schweizeri* and the other taxa may be at least partly attributed to the diverse methodologies applied.

Among the venoms of *M. lebetina* subspecies, some differences can be detected. For instance, vascular endothelial growth factors (VEGF) and Kunitz-type inhibitors (KUN) were found only in one of the four *M. l. obtusa* venom proteomes, and KUN was detected only in the most recent *M. l. transmediterranea* venom composition. Additionally, the two *M. l. transmediterranea* venom proteomes lack l-amino acid oxidases (LAAO), cysteine-rich secretory proteins (CRISP), and all of the secondary and rare toxin families identified in the other taxa (Table 1). Given that both *M. l. transmediterranea* venom proteomes were obtained from the venom of a captive specimen of reportedly undefined origin^109,112^, doubts have been raised regarding the accuracy of its identification^36,116^. For instance, considering that this taxon has not been observed in North Africa for decades, it seems possible that the analysed venom was obtained from a *M. lebetina* individual not originating from North Africa, and therefore belonging to a different subspecies. Furthermore, the relative abundances of most toxin families within the analysed *M. l. transmediterranea* venom differ considerably from those reported for other *Macrovipera* venoms, and are instead more similar to those of the toxin families identified in the venom of *D. mauritanica*^11^ (Table 1). This large viper species is widely distributed across North Africa and is morphologically similar to snakes of the genus *Macrovipera*, within which it has sometimes been included as *Macrovipera mauritanica*^52^. While we do not advocate deriving taxonomic inferences primarily from similarities and differences in proteomic venom profiles, in light of the above considerations, we do not exclude the possibility that the analysed *M. l. transmediterranea* venom may, in fact, have been obtained from a misidentified *D. mauritanica* individual.

A recent comparison of the chromatographic and electrophoretic venom profiles of *M. schweizeri*, *M. l. lebetina*, *M. l. cernovi*, *M. l. obtusa* and *M. l. turanica* further highlighted their general similarity^36^. Nonetheless, several discrepancies in the intensities of peaks and bands suggest the presence of quantitative differences between and within taxa. For instance, although svMP is the predominant toxin family in all analysed venoms, its relative abundance varies, ranging from 22% in *M. l. obtusa*^115^ to 67% in *M. l. transmediterranea*^109^. Similarly, the relative abundance of svSP, another major component of blunt-nosed viper venoms, ranges between 6% in *M. l. transmediterranea*^112^ and 24% in *M. l. obtusa*^113^. Furthermore, considerable qualitative and quantitative differences were observed between the protein profiles of *M. l. obtusa* and *M. l. lebetina* venoms obtained via two-dimensional polyacrylamide gel electrophoresis (2D-PAGE)^111^. A study comparing the venoms of these two subspecies using Fourier-transform infrared spectroscopy also highlighted the presence of spectral differences between them^117^. Additionally, a recent study revealed that *M. l. obtusa* venoms from a restricted region in south-eastern Anatolia (Türkiye) may exhibit individual variation in terms of protein profiles and enzymatic activities^118^. In light of the above, and considering that variation in snake venoms occurs at every taxonomic level as well as at the individual level^10,119–121^, it appears reasonable to hypothesise that venomics analyses conducted at a finer scale may reveal a greater degree of variability in *Macrovipera* venoms than currently anticipated. This could be particularly relevant in the context of potential ontogenetic dietary changes in blunt-nosed vipers. Indeed, several snake species exhibit varying degrees of age-related differences in venom composition^119,122,123^, often associated with dietary shifts during growth (e.g., from an ectotherm-based to an endotherm-based diet). Although little is known about the feeding ecology of members of the genus *Macrovipera*, evidence suggests that juvenile *M. schweizeri* primarily prey on ectotherms (i.e., lizards), before transitioning to an avian-based diet in adulthood^91^. In light of this, while no studies have directly compared the venom compositions of juvenile and adult *Macrovipera*, it is plausible to hypothesise that differences may exist, at least in this taxon.

## Biological activity and pharmacological potential

Over the years, several studies have investigated the biological activities of various crude venoms and purified proteins/peptides of the genus *Macrovipera*. Using both in vivo and in vitro methodologies, these works aimed to elucidate the relationship between venom composition and function in blunt-nosed vipers. Generally, their venoms exert potent haemorrhagic, cytotoxic, and coagulopathic activities, consistent with the high abundance of svMP, PLA_2_, svSP and CTL^124–127^ (Table 1). Furthermore, several isolated compounds have been found to inhibit platelet aggregation (e.g., lebecetin^128^) and angiogenesis (e.g., lebestatin^129^), or induce fibrinogenolytic and haemorrhagic effects^130,131^. In light of the potency of *Macrovipera* venoms, and considering that animal venoms can constitute a prolific source of biomedical innovation^132,133^, substantial effort has been made to evaluate the powerful toxins of this genus for pharmacological use^40,41^. As detailed information on the pharmacology and bioactivity of individual components of *Macrovipera* venoms has been recently provided by Siigur and colleagues^41^, this section presents an overview of the biological activities of blunt-nosed viper crude venoms in various human cell lines and other systems (resumed in Table 2), as well as insights into selected components holding particularly high translational potential.

**Table 2:** Targets for assessing the biological activity of *Macrovipera* crude venoms

^142^Target	*M. l. lebetina*	*M. l. cernovi*	*M. l. obtusa*	*M. l. turanica*	*M. schweizeri*		References
**Human**							
CaCo-2 (colon adenocarcinoma)			✓				^39^
HEK293T (Human Embryonic Kidney 293 variant)		✓	✓	✓	✓		^36^
HUVEC (Human Umbilical Vein Endothelial Cells)	✓^β, γ^	✓^γ^	✓^α, β,γ^	✓^γ^	✓^γ^		^143^^α^; ^142^^β^, ^36^^γ^
K562 (chronic myelogenous leukaemia)			✓				^141^
MCF-7 (breast cancer)			✓				^39^
MDA-MB-231 (breast cancer)		✓	✓	✓	✓		^36^
U-87 MG (malignant glioblastoma)			✓				^39^
Plasma	✓	✓	✓	✓	✓		^35^
nAChR (nicotinic acetylcholine receptor) mimotopes	✓	✓	✓	✓	✓		^144^
**Monkey**							
Vero (*Chlorocebus sabaeus* kidney)			✓				^39^
**Mouse**							
L929 (fibroblast cells)	✓						^34^
RAW264.7 (macrophage)		✓	✓	✓	✓		^36^
Liver, kidney, lung, and heart tissues			✓				^136^;^137^
**Rat**							
Neonatal cardiomyocytes and nonmyocytes			✓				^138^
Atria and perfused mesenteric bed			✓				^145^*
Paw oedema model			✓				^139^
**Guinea pig**							
Ileum			✓				^145^*
**Gram-positive bacteria**							
*Bacillus cereus* ATCC 7064			✓				^39^
*Enterococcus faecalis* ATCC 29212			✓				^39^
*Enterococcus faecium* DSM 13590			✓				^39^
*Staphylococcus aureus* ATCC 25923		✓^β^	✓^α,β^	✓^β^	✓^β^		^39^^α^; ^36^^β^
*Staphylococcus epidermidis* ATCC 12228		✓^β^	✓^α,β^	✓^β^	✓^β^		^39^^α^; ^36^^β^
*Listeria monocytogenes* DSM 20600		✓	✓	✓	✓		^36^
**Gram-negative bacteria**							
*Escherichia coli* ATCC 25922		✓^β^	✓^α,β^	✓^β^	✓^β^		^39^^α^; ^36^^β^
*Escherichia coli* 0157:H7			✓				^39^
*Proteus vulgaris* ATCC 6957			✓				^39^
*Salmonella typhimurium* CCM 5445			✓				^39^
**Fungi**							
*Candida albicans* ATCC 10239			✓				^39^
**Artificial**							
Giant unilamellar vesicles (GUV)			✓				^134^; ^135^

The table provides information on the tested targets, categorised by type, along with the *Macrovipera* taxon from which the analysed venom was obtained, and the corresponding references. In the case of human nAChR mimotopes, those from amphibians, birds, lizards and rodents (rats) were also tested. An asterisk (*) indicates publications where the assignment of the *M. lebetina* subspecies from which the analysed venom was sourced is uncertain.

### Biological activities of crude venoms

Among the *Macrovipera* taxa currently recognised, *M. l. obtusa* is arguably the most investigated. For instance, Ayvazyan and Ghazaryan^134^ and Ayvazyan et al.^135^ tested the effect of *M. l. obtusa* venom on giant unilamellar vesicles (GUV) of mixed-lipids, and observed morphological changes such as distortions in the vesicle membrane and increased vesicle size. Similarly, the authors showed that *M. l. obtusa* venom increases the electrical resistance of bilayer lipid membranes from rat liver and muscle lipids, and significantly decreases GUV membrane fluidity. More recently, Yücel Ağan and Hayretdağ^136^ observed histopathological effects of *M. l. obtusa* venom from Türkiye on liver, kidney and heart tissues of mice, such as cellular degeneration, mononuclear cell infiltration, haemorrhage, and necrosis, indicating systemic harm. Similar effects were described for Armenian *M. l. obtusa* venom^137^, along with dose-dependent effects on attachment and metabolic activity of rat neonatal cardiomyocytes and nonmyocytes, with immediate cytotoxic effects at a relatively high concentration of 100 μg/ml^138^. Furthermore, *M. l. obtusa* venom from south-eastern Türkiye was shown to induce inflammation in a rat paw oedema model^139^, and venoms from juvenile and adult individuals from Georgia exhibited similarly strong procoagulant properties, potently activating multiple blood clotting factors in human plasma (i.e., Factor VII, Factor X and Factor XII)^140^.

Furthermore, Ozen et al.^39^ investigated the cytotoxic and antimicrobial activities of *M. l. obtusa* venom against various human cancer cells, Gram-negative and Gram-positive bacteria, and the fungus *Candida albicans*. The venom inhibited cancer cell proliferation in a dose-dependent manner, and showed selective activity among different human cancer cell lines, such as U-87 MG (malignant glioblastoma), MCF-7 (breast cancer) and CaCo-2 (colorectal adenocarcinoma); half maximal inhibitory concentration (IC_50_) values: 1.90, 3.85 and 4.75 μg/ml, respectively. Nonetheless, potent cytotoxic activity was observed also against non-human, non-cancerous cells (i.e., Vero, *Chlorocebus sabaeus* renal epithelial cells; IC_50_ value: 1.18 μg/ml). While moderate antifungal activity was observed against *C. albicans*, with a minimum inhibitory concentration (MIC) of 62.50 μg/ml, no strong inhibition on Gram-negative and Gram-positive bacteria was detected. In vitro cytotoxicity tests of the venom from this subspecies against K562 (human chronic myelogenous leukaemia) cells showed dose-dependent toxicity at various concentrations after 72 h^39^. Nuclear fragmentation and condensation, apoptotic bodies, and activation of caspase-3 (indicative of apoptosis induction) were also observed^141^.

A comparative analysis of *M. l. obtusa* and *M. l. lebetina* venoms by İğci et al. revealed dose- and time-dependent cytotoxicity on human umbilical vein endothelial cells (HUVEC)^142^. At the highest concentration (24 μg/ml), *M. l. obtusa* and *M. l. lebetina* venoms reduced cell viability by 46% and 49% (3-h treatment), and by 68% and 65% (16-h treatment), respectively. After 24 h of treatment, cell viability was reduced by 73% for both venoms. The IC_50_ values for the 24-h treatment were 7.32 and 6.28 μg/ml for *M. l. obtusa* and *M. l. lebetina* venoms, respectively. The venom of Iranian *M. lebetina* from an unspecified locality of origin was found to induce dose-dependent cytotoxicity in HUVEC, with an IC_50_ value of 11.77 μg/ml after 24 h of incubation^143^. The cytotoxic effect and IC_50_ value of *M. l. lebetina* venom on mouse fibroblastic cells (L929) were also investigated by Nalbantsoy et al. ^34^ The authors reported dose-dependent inhibitory effects on cell proliferation (IC_50_ value after 2-h treatment: 1.62 μg/ml; after 48-h treatment: 0.62 μg/ml) and various morphological abnormalities in the tested cells. The LD_50_ value measured in mice was tenfold higher than the IC_50_ value^34^.

Chowdhury et al.^35^ investigated the venoms of *M. l. lebetina*, *M. l. cernovi*, *M. l. obtusa*, *M. l. turanica* and *M. schweizeri*, and detected strong procoagulant effects on human plasma for all of them at the tested concentrations (from 0.25 to 50 μg/ml). The five venoms showed extremely potent clotting-promoting activity, rapidly forming strong, stable clots with varying degrees of Factor X activation and overall low dependence on phospholipids. For the same venoms, toxin binding on the α1 neuronal nicotinic acetylcholine receptors (nAChRs) orthosteric sites of amphibian, avian, lizard, rodent (rat), and human mimotopes was tested. All *M. lebetina* subspecies presented higher affinity for amphibian mimotopes, whereas *M. schweizeri* showed stronger relative binding to lizard mimotopes. Furthermore, the *M. lebetina* subspecies also presented considerable affinity for the human mimotope^144^. It is worth mentioning that earlier studies on the venom of an undefined *M. lebetina* subspecies of Iranian origin reported neuromuscular blockade, haemodynamic alterations, and cardiovascular collapse in the animal models considered^145^.

In line with previous works^34,39,142^, the analysis by Schulte et al. on the venoms of *M. l. cernovi*, *M. l. obtusa*, *M. l. turanica* and *M. schweizeri* showed potent concentration-dependent cytotoxic activities against HEK293T and RAW264.7 cells^36^. The highest venom concentration (25 μg/ml) caused a reduction in cell viability of ~75%. Interestingly, almost identical activities were measured against HUVEC, MDA-MB-231, HEK293T and RAW264.7 cells. Similarly, comparable proteolytic activity was detected among most taxa. Only *M. l. obtusa* displayed marginally higher activity, especially at a concentration of 100 μg/ml, suggesting differences in the quantity or potency of proteolytic enzymes among subspecies. Contrary to what was reported by Ozen et al.^39^, the tested venoms exerted notable antimicrobial effects against several bacterial strains even at lower concentrations (Table 2).

### Venom components with pharmacological potential

**Fig. 3:**
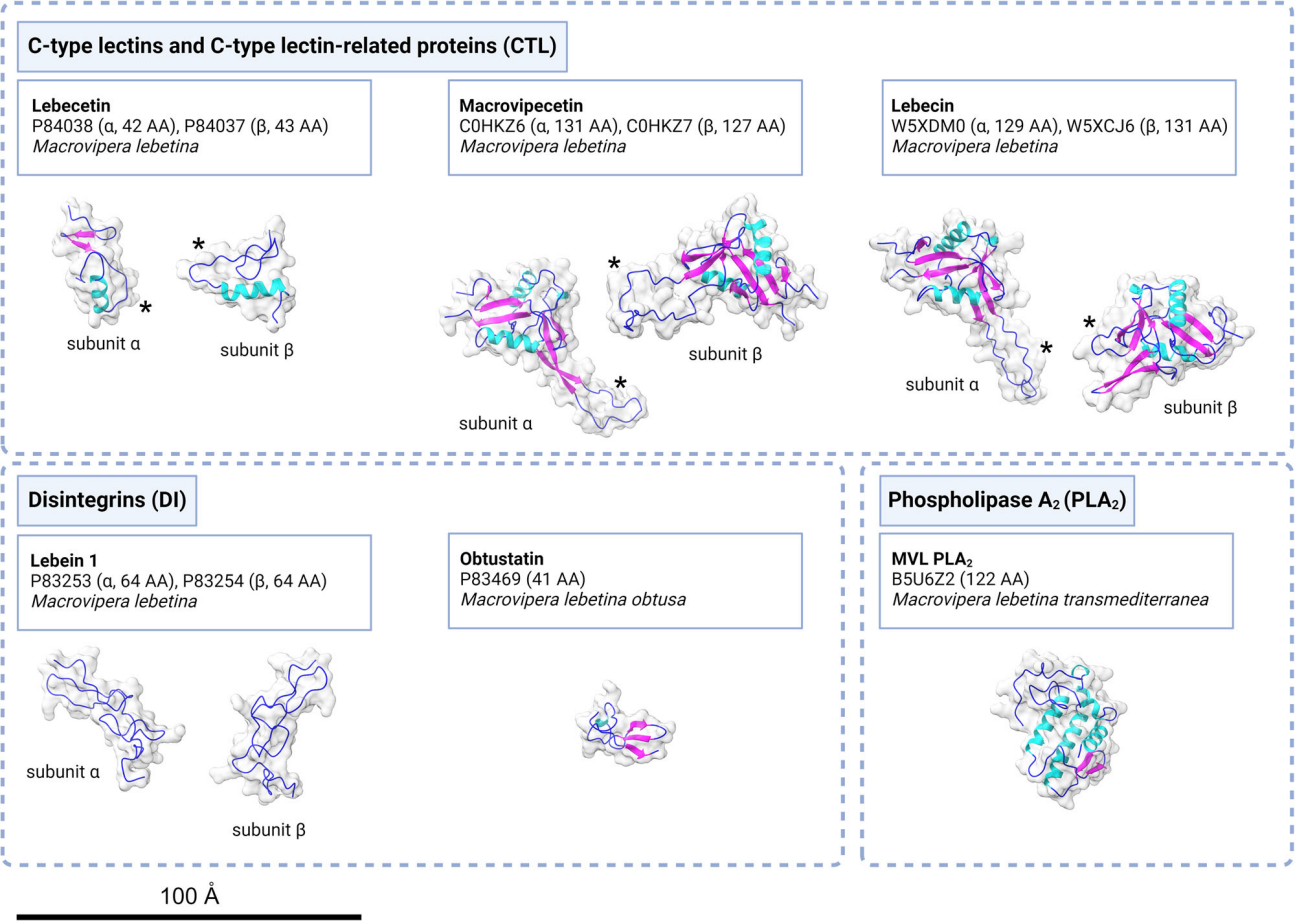
Molecular structure of selected *M. lebetina* venom proteins with anticancer potential. The surface and secondary structures of the mature proteins are shown, with helices in cyan, sheets in magenta, and loops in blue. UniProt sequence identifiers, taxon of origin, and number of amino acids are listed below the component names. For CTLs, the asterisks (*) indicate the index finger loop-swap domain, including the long loop and bay region^125^. The protein structures were modelled using AlphaFold 3^204^ and illustrated using ChimeraX^205^. Additional details are provided in Table S2. Figure assembled using BioRender (https://www.biorender.com).

As one of the primary effects caused by *Macrovipera* venoms is tissue damage^137,146^, venom toxins from blunt-nosed vipers have been particularly studied for targeted therapeutic applications where these effects could be exploited. Indeed, bioactivity profiling of crude *Macrovipera* venoms has indicated their potential for developing novel anticancer agents^36,41^. This suggests that a variety of previously unidentified biomolecules with activity against cancer cells could be isolated from these venoms. Therefore, most biomedical research has focused on toxin families with cytotoxic effects, including CTL, DI, and PLA_2_ (see Fig. 3), although other components, such as serine protease inhibitors, have also been found to exhibit potent antineoplastic properties^147^. However, despite the numerous studies investigating or reporting the anticancer properties of *Macrovipera* venom components, these findings should be considered preliminary and require further experimental validation.

Ca^2+^-dependent CTL are common in snake venoms, have a molecular weight of ~30 kDa, and constitute major components of viperine venoms^11,126^. They cause an array of symptoms, mainly involving coagulotoxicity, alteration of platelet aggregation and tissue damage. CTLs can inhibit tumour growth, invasion and metastasis by disrupting cell–cell and cell–matrix interactions, thereby interfering with cell adhesion. They can also induce apoptosis in cancer cells by modulating intracellular signalling pathways, leading to programmed cell death. Furthermore, their anti-angiogenic properties hinder the formation of new blood vessels, depriving tumours of the nutrients and oxygen required for growth^126^. The pharmacological properties of several CTLs from *Macrovipera* venoms have been evaluated. Lebecetin is a disulphide-linked heterodimeric CTL of 31 kDa isolated from *M. lebetina* venom^128,148^. This toxin impacts the adhesion capacity of cancer cells (IGR39 melanoma and HT29D4 adenocarcinoma), demonstrating particularly strong effects on fibrinogen and laminin, but not on fibronectin or type I and IV collagen^128,148^. Additionally, macrovipecetin and lebecin, dimeric CTLs recently isolated from *M. lebetina* venom, have been suggested to possess anticancer potential. Macrovipecetin was shown to potently reduce the viability of SK-MEL-28 melanoma cells in vitro, and this effect could be enhanced when combined with cisplatin treatment^149^. The synergistic combination of macrovipecetin and cisplatin increased the apoptotic rate of tested melanoma cells^149^. Lebecin has been found to potently inhibit the proliferation of MDA-MB231 human breast cancer cells and their integrin-mediated attachment to substrates in a dose-dependent manner^150^.

In addition to lectins and lectin-like proteins, a range of other *Macrovipera* toxins has been investigated for potential anticancer effects. For instance, disintegrins specifically bind to integrins in cancer cells, thereby blocking signalling pathways that promote survival, proliferation, migration, and invasion. By inhibiting integrin-mediated cell adhesion to the extracellular matrix, disintegrins prevent cancer cells from detaching, migrating, and invading new tissues, thus inhibiting metastasis^151^. Additionally, disrupting integrin signalling can induce apoptosis, leading to the elimination of cancer cells^152^. One of the disintegrins investigated for anticancer potential is obtustatin, a monomeric disintegrin (DI) of approximately 4.4 kDa isolated from the venom of *M. l. obtusa*^153^. Obtustatin was determined to be a potent inhibitor of α1β1 integrin with high selectivity, inhibiting, for instance, angiogenesis and tumour development in K562 and lung carcinoma cells^154^. Lebein, another DI isolated from *M. lebetina* venom (~14 kDa), also showed anticancer potential, exerting apoptosis-inducing effects on melanoma and colon cancer cells, as well as antiplatelet activity^155,156^.

Another abundant component of *Macrovipera* venoms is PLA_2_, a toxin family also often associated with cytotoxic and anticancer activities^157,158^. One of the PLA_2_s from this genus is MVL-PLA_2_, an acidic Asp49 PLA_2_ of 13.6 kDa isolated from *M. l. transmediterranea* venom^159^. This toxin did not show cytotoxicity when administered below 2 µM, but inhibited the growth and adhesion of multiple tumour cells^159^. Subsequent chemical modification with p-bromophenacyl bromide resulted in the inactivation of the enzymatic activity of MVL-PLA_2_ without impacting its anticancer effects, suggesting that its PLA_2_ activity is not involved in the exerted effect against cancer cells^159^.

## Clinical symptoms of envenomation

**Fig. 4:**
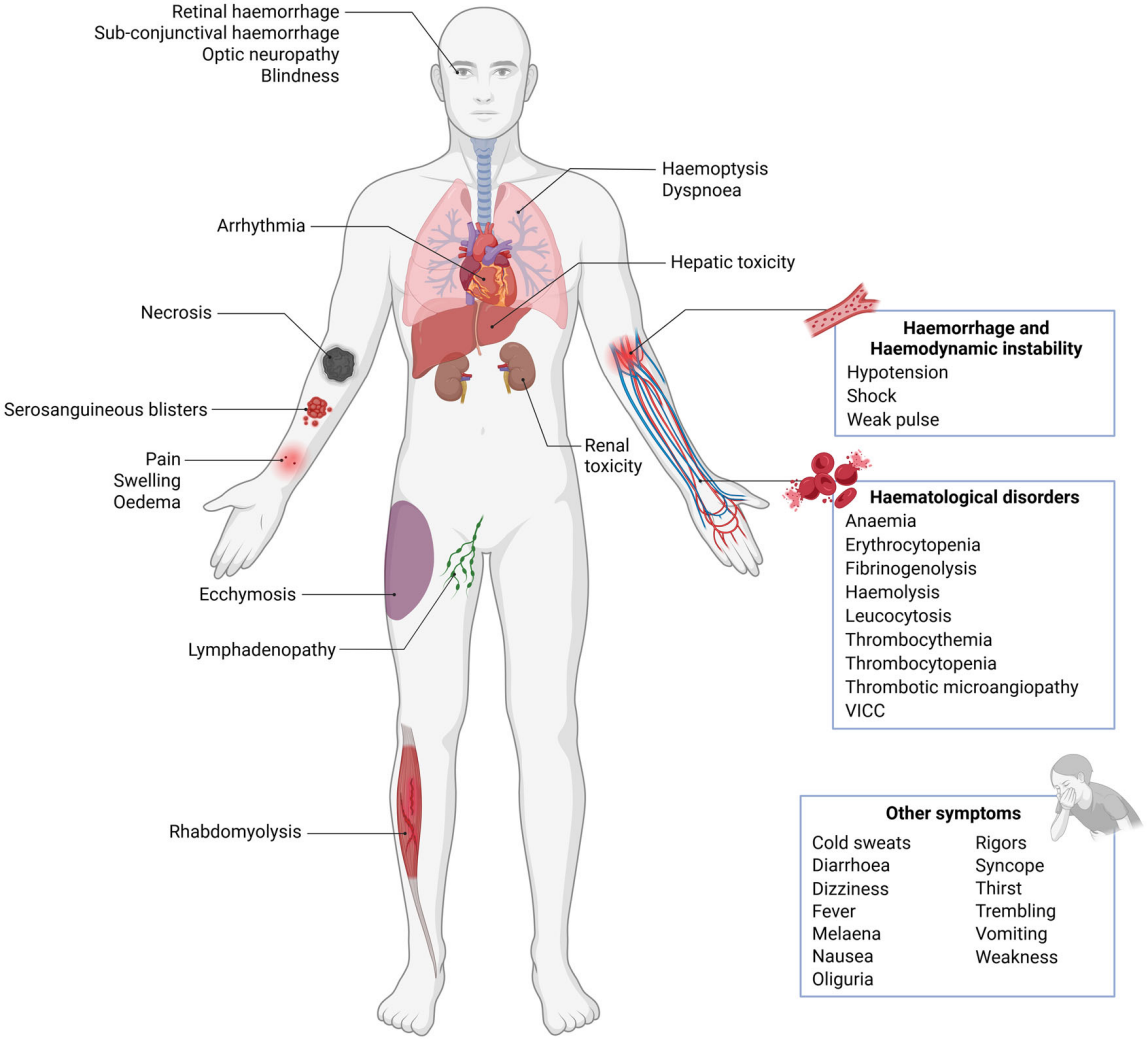
Clinical symptoms associated with *Macrovipera* envenoming. The diagram resumes the effects described in blunt-nosed viper bite reports available in the literature. Additional details are provided in Table S1. Figure assembled using BioRender (https://www.biorender.com).

Viperid envenoming is notorious for causing severe clinical symptoms, typically of haemorrhagic and cytotoxic nature^14,160^, although neurotoxicity has also been reported for a number of species^161,162^. Swelling, pain, blisters and necrosis are among the most common local effects of viperid envenoming, whereas systemic effects include potentially life-threatening symptoms such as haemorrhage, thrombocytopenia, and venom-induced consumption coagulopathy (VICC)^163,164^. The clinical effects observed after envenoming by snakes of the genus *Macrovipera* follow this general trend (Fig. 4), and align with the compositional and functional profiles of their venoms. Based on the envenomation reports published to date^83,146,165–180^ (see Table S1), we hereby provide a holistic overview of the clinical symptoms elicited by *Macrovipera* bites.

Following envenomation, local symptoms can develop within 15 min from the bite, with pain and swelling being the first to appear^170,181^. Swelling can rapidly extend from the bite site to the whole bitten limb and the trunk, typically followed by bruising and ecchymosis. Serosanguineous blisters and subsequent tissue necrosis are common, and often require amputation when involving the digits^31,146,172,181^. Compartment syndrome may arise, and can have systemic consequences if not treated promptly^146,160^.

Haematological alterations are among the most severe and common systemic manifestations of *Macrovipera* envenoming (Table S1). The combination of coagulopathy, thrombocytopenia, and haemolysis can result in severe anaemia^176,178^. Thrombotic microangiopathy, rhabdomyolysis, VICC and hepatic and renal toxicity may develop in the more severe cases^181,182^. Among systemic symptoms frequently occurring after blunt-nosed viper bites are fever, cold sweats, dizziness, syncope, thirst, nausea, vomiting, diarrhoea, rigors and trembling^31,181^. Neurotoxic symptoms have rarely been reported for *Macrovipera* bites, with some evidence suggesting weakness and somnolence^175^, as well as possible polyneuropathy^169^.

It should be noted that the vast majority of the symptoms reported above have been described exclusively for *M. lebetina*. Indeed, the first literature records of *M. razii* snakebite were only recently published, and describe symptoms ranging from swelling, itchiness, and necrosis at the bite site to haemodynamic disturbances^180^. However, details regarding the medical evaluations conducted are not provided. Similarly, only two case reports concerning envenomation caused by *M. schweizeri* currently exist. Pain and extensive swelling, increased blood sugar, thrombocythemia, erythrocytopenia, hypotension and bradycardia were the symptoms described in the first case^83^, while a more recent study reports fibrinogenolysis and VICC in a subject bitten by a captive specimen^179^. Considering that, at equal concentration, the biological activity of *M. schweizeri* venom is comparable to that of other *Macrovipera* taxa^36^, the relatively milder severity of *M. schweizeri* envenomation symptoms may be attributable to a lower venom yield in this generally smaller species.

Despite the limited number of published bite reports, the severity of the symptoms and the potentially fatal outcome of *Macrovipera* envenoming have led the WHO to classify this genus as of the highest medical importance (Category 1) in 13 of the 20 countries within its distribution range^30^. Historically, blunt-nosed vipers have been reported as the leading cause of snakebite fatalities in Iraq, with a 50% mortality rate among the bitten subjects^183^. In contrast, a recent analysis of 1122 *M. l. obtusa* bite cases that occurred in Azerbaijan between 2009 and 2020 revealed a case-fatality rate of just 1.9%^184^. The fragmented nature of snakebite data from most countries within the *Macrovipera* range complicates the validation and comparison of these estimates^23,32^.

## Snakebite management and treatment

The distribution of *Macrovipera* envelops some of the most rural, remote areas of the Palaearctic region, where hospitals and health stations may be difficult to reach and are often not properly equipped for the treatment of snakebite^185^. In this scenario, educating local communities to prevent snakebite accidents caused by blunt-nosed vipers is crucial. For instance, understanding the activity patterns and preferred habitats of these snakes would aid in avoiding them, thereby reducing the risk of human-snake encounters. Activities such as dislodging logs or boulders with bare hands, or inserting sticks or fingers into burrows, holes and crevices, are dangerous. Furthermore, the use of protective clothing (e.g., shoes, boots and long trousers), along with carrying a light at night, especially when walking on unlit paths, is recommended to minimise the risk of bites. Practices involving disturbing and/or cornering snakes should be avoided at all times, and handling should be only performed by professionals^181^.

In the case of snakebites, guidelines recommend that bitten individuals should be transported swiftly and with minimal movement to the nearest facility capable of providing urgent medical care^20,186,187^. When this is not immediately available, effective first aid can extend the patient’s survival until they reach professional medical care. Focusing on *Macrovipera* envenoming, we hereby report the principles of snakebite management and treatment and discuss the antivenom therapy available against this genus.

### First aid

The three principles of first aid are: (i) rapid transportation of the snakebite victim to a medical facility; (ii) delaying the progression of severe envenoming until professional care is available; (iii) mitigating severe and potentially life-threatening initial symptoms of envenoming^181,186^. The bitten subject should be reassured to prevent tachycardia and anxiety, which could exacerbate envenoming by accelerating blood flow and facilitating the spread of venom throughout the body. Reassurance can be based on the understanding that not all bites result in envenoming (i.e., 'dry bites'), that envenoming typically progresses slowly (sometimes over days^146,178^), that the overall case-fatality rate is low, and that modern medical treatments are highly effective^160,181^.

It is advisable to clean the bite area with water and alcohol-free detergents, remove any constricting accessories such as bracelets and watches, and immobilise the affected limb with an improvised splint or sling. The affected limb should remain immobile, as muscle contractions will facilitate the systemic absorption of venom. Outdated and potentially harmful practices, including local cauterisation, incision, excision, amputation, mouth suction, the use of vacuum pumps or syringes, tourniquets, traditional remedies like herbs and infusions, and the application of electric shocks or ice, must be avoided^20,181^. In particular, the application of tourniquets can be especially harmful in cases of snakebites expected to induce strong cytotoxic effects, such as those from *Macrovipera* (see Table S1). Indeed, tourniquets may exacerbate tissue damage by restricting blood flow to the affected area, possibly leading to ischaemia, necrosis, and gangrene of the surrounding tissues^188^. Furthermore, pressure bandages, which are typically recommended as first aid for snakebites, can be harmful in cases involving predominantly cytotoxic effects and should, therefore, be avoided. If the offending snake has been killed, it is advisable to collect it (cautiously and without heightening the risk of further bites) and hand it over to professionals for identification. This can be useful in determining the most appropriate treatment to be administered by medical personnel. However, it is essential to avoid handling the snake with bare hands, even if dead, to prevent the risk of further bites. In most cases, photographing the snake that inflicted the bite provides an adequate alternative for identification.

### Management of early symptoms

Symptoms of *Macrovipera* envenoming can manifest within minutes or may take several hours to appear. Therefore, it is recommended that snakebite victims be monitored in a hospital setting for a minimum of 24 h following the bite. Pain, alteration of heart rate and rhythm, nausea, vomiting, abdominal pain and diarrhoea are among the most common early symptoms of envenomation (see section 'Clinical symptoms of envenomation' and references therein). Persistent pain in the affected area can be managed with analgesic therapy, while avoiding acetylsalicylic acid (aspirin), which inhibits thrombocyte function and could thus precipitate bleeding in patients with severe envenomation. Prophylactic antibiotic therapy should be prescribed only in cases of proven secondary risk of infection (e.g., extensive necrosis or dirty wounds). Tetanus immunisation status should always be evaluated, and immunisation provided as needed. The treatment of internal complications (e.g., hepatotoxicity, nephrotoxicity, and cardiotoxicity) does not differ from that of aforementioned complications of different aetiologies.

Hypotensive patients should be placed in the head-down position. Vomiting, a common early indicator of severe systemic envenomation, can be addressed by giving an antiemetic (e.g., ondansetron, promethazine and dimenhydrinate) at the standard dosage, tailored to the individual patient’s needs and clinical condition. In rare instances, individuals previously bitten or otherwise exposed to specific snake venom may exhibit immediate type I hypersensitivity upon subsequent bites. Treatment for these anaphylactic reactions adheres to the established guidelines for managing anaphylaxis from other causes.

Although treatment should always be administered by professional medical personnel, nurses, paramedics, and community first aid workers can be trained to perform tasks such as positioning snakebite victims properly and administering medications.

### Antivenom therapy

To date, snake antivenom is the only specific antidote to the toxins found in snake venom^189^. Snake antivenom is produced starting from hyperimmune serum collected from a large mammal (typically a horse), which has been injected with increasing doses of venom over several months. This process results in the production of progressively rising neutralising antibodies (immunoglobulins G, or IgG) that are specific to the venoms used for immunisation^190^. In light of this, and considering that venom variation is to be expected^10^, it stands to reason that different antivenoms are most often required to effectively tackle different venom compositions. Nonetheless, there is evidence of antivenoms neutralising venoms of other species than those included in the immunisation scheme (i.e., paraspecificity, or cross-neutralisation)^191,192^.

Considering the threat vipers of the genus *Macrovipera* pose in terms of snakebite, particularly in the Near and Middle East^23,31,32^, a number of antivenoms have been developed over the years to neutralise their venoms. However, remarkably little information about these antivenoms is available from manufacturers’ websites, and the details reported in online databases can sometimes be misleading. For instance, while the WHO Snakebite Information and Data Platform^30^ suggests that *Macrovipera razii* venom may have been included in the immunisation mixtures used for the development of the antivenoms produced by the Razi Vaccine & Serum Research Institute (Iran) and Padra Serum Alborz (Iran), this has been recently disproved^74,193^. In light of this situation, Table 3 provides updated, cross-checked information on the antivenoms against *Macrovipera* venoms currently listed by the WHO.

**Table 3:** Antivenoms against *Macrovipera* venoms listed by the WHO

Antivenom name	Manufacturer	Country	IgG type [source]	Specificity
**European viper venom antiserum**	Imunološki Zavod	Croatia	F(ab')_2_ [equine]	*Vipera ammodytes*; manufacturer label claim of paraspecificity: ***Macrovipera lebetina***, *Montivipera xanthina*, *Vipera aspis*, *Vipera berus*
**Gamma-Vip**	Institut Pasteur de Tunis	Tunisia	F(ab')_2_ [equine]	***Macrovipera lebetina***, *Cerastes cerastes*
**Hexavalent snake venom immunoglobulin**	Razi Vaccine & Serum Research Institute	Iran	F(ab')_2_ [equine]	***Macrovipera lebetina cernovi***, *Echis carinatus sochureki*, *Montivipera raddei*, *Gloydius caucasicus*, *Naja oxiana*, *Pseudocerastes persicus*
**Inoserp™ Europe**	Inosan Biopharma S. A.	Spain	F(ab')_2_ [equine]	***Macrovipera lebetina cernovi***, ***Macrovipera lebetina obtusa***, ***Macrovipera lebetina turanica***, ***Macrovipera schweizeri***, *Vipera ammodytes, Vipera aspis, Vipera berus, Vipera latastei, Montivipera xanthina*
**Inoserp™ MENA**	Inosan Biopharma S. A.	Spain	F(ab')_2_ [equine]	***Macrovipera lebetina***, *Bitis arietans*, *Cerastes cerastes*, *Echis leucogaster*, *Montivipera raddei*, *Naja haje*, *Naja nigricollis*, *Naja pallida*, *Pseudocerastes fieldi*, *Pseudocerastes persicus*, *Walterinnesia aegyptia*; manufacturer label claim of paraspecificity: ***Macrovipera lebetina obtusa***, ***Macrovipera lebetina transmediterranea***, ***Macrovipera lebetina turanica***, *Cerastes gasperettii*, *Cerastes vipera*, *Daboia mauritanica*, *Daboia palaestinae*, *Echis carinatus sochureki*, *Echis coloratus*, *Echis khosatzkii*, *Echis megalocephalus*, *Echis omanensis*, *Echis pyramidum*, *Montivipera bornmuelleri*, *Montivipera raddei kurdistanica*, *Naja nubiae*, *Naja oxiana*, *Vipera latastei*
**IPAVIP Antiviperin Sera**	Institut Pasteur d’Algerie	Algeria	IgG2a [equine]	***Macrovipera lebetina***, *Cerastes cerastes*
**Monovalent serum against snake venom gyurza**	UzbioPharm LLC	Uzbekistan	F(ab')_2_ [equine]	***Macrovipera lebetina***
**Pentavalent snake antivenom immunoglobulin**	Razi Vaccine & Serum Research Institute	Iran	F(ab')_2_ [equine]	***Macrovipera lebetina cernovi***, *Echis carinatus sochureki*, *Montivipera raddei*, *Gloydius caucasicus*, *Pseudocerastes persicus*
**Polisera**	Vetal Serum ve Biyolojik Ürünler Üretim Sanayi ve Ticaret A.Ş.	Türkiye	F(ab')_2_ [equine]	***Macrovipera lebetina***, *Montivipera xanthina*, *Vipera ammodytes*
**Polyvalent Anti-Snake Serum**	Egyptian Organisation for Biological Products and Vaccines (VACSERA)	Egypt	F(ab')_2_ [equine]	*Cerastes cerastes*, *Naja haje*, *Naja nubiae*; manufacturer label claim of paraspecificity: ***Macrovipera lebetina***, *Bitis arietans*, *Bitis gabonica*, *Cerastes vipera*, *Daboia palaestinae*, *Echis coloratus*, *Echis carinatus*, *Naja melanoleuca*, *Naja mossambica*, *Naja nigricollis*, *Naja oxiana*, *Naja pallida*, *Pseudocerastes persicus*, *Vipera ammodytes*, *Walterinnesia aegyptia*
**Polyvalent Anti-Vipers Serum**	Egyptian Organisation for Biological Products and Vaccines (VACSERA)	Egypt	F(ab')_2_ [equine]	*Cerastes cerastes*, *Echis coloratus*, *Echis pyramidum*; manufacturer label claim of paraspecificity**:** ***Macrovipera lebetina***, *Cerastes vipera*, *Echis carinatus*, *Montivipera xanthina*, *Pseudocerastes fieldi*, *Vipera ammodytes*
**Polyvalent serum against snake venoms gyurza, efa, and cobra**	UzbioPharm LLC	Uzbekistan	F(ab')_2_ [equine]	***Macrovipera lebetina***, *Echis carinatus*, *Naja oxiana*
**SnaFab5**	Padra Serum Alborz	Iran	F(ab')_2_ [equine]	***Macrovipera lebetina cernovi***, *Echis carinatus sochureki*, *Gloydius caucasicus*, *Montivipera raddei*, *Pseudocerastes persicus*
**SnaFab6**	Padra Serum Alborz	Iran	F(ab')_2_ [equine]	***Macrovipera lebetina cernovi***, *Echis carinatus sochureki*, *Gloydius caucasicus*, *Montivipera raddei*, *Naja oxiana*, *Pseudocerastes persicus*

For each antivenom, the table provides the name, manufacturer, country of production, type and source of IgG, and immunising/neutralised snake species. *Macrovipera* taxa are reported in bold. Information was gathered from the WHO Snakebite Information and Data Platform^30^, the Snakebite Envenoming Medicines Database^194^, Dalhat et al.^203^, Dehghani et al.^31^, Alshalah et al.^32^, and Jalali et al.^193^.

*IgG* immunoglobulin G; *F(ab')*_*2*_ antibody fragment with two antigen-binding sites including part of the hinge region of the antibody.

The WHO also lists Viekvin, an antivenom produced by the Institute of Virology, Vaccines and Sera, TORLAK (Serbia), as effective against *M. lebetina*^30^. Nonetheless, the patient information leaflet for this product (available from https://torlak.rs/en/production/serums) clearly states that it is ineffective against the venoms of snake species other than *Vipera ammodytes* and *Vipera berus*, and therefore we did not include it in Table 3. Another antivenom, named Antivenom-2 Polyvalent Anti-Snake Venom Sera, and manufactured by the Scientific Studies & Research Centre (Syria) against the venoms of *Macrovipera lebetina*, *Daboia palaestinae* and *Vipera ammodytes*, has been used until recent times in Lebanon, but is currently very difficult to obtain^31^.

The Snakebite Envenoming Medicines Database^194^ lists one additional antivenom purportedly effective against *Macrovipera* venoms, namely Menaven, which is not included by the WHO among the antivenoms currently available for this genus^30^. This antivenom, also known as Biosnake, is produced by VINS Bioproducts Ltd (India) from purified equine plasma hyperimmunised against the venoms of *Cerastes cerastes*, *Naja haje* and *Naja nigricollis*. Notably, the manufacturer claims that Menaven/Biosnake can neutralise the venoms of 12 additional medically relevant snake species from Africa, Europe and the Middle East, including *M. lebetina*. However, recent evidence indicates that Menaven/Biosnake is clinically ineffective against snakebites from *M. lebetina* and other medically relevant viperids, despite the manufacturer’s claim of paraspecificity^177^.

A number of new, promising anti-*Macrovipera* antivenoms are currently being produced and tested. A novel antivenom, MENAVip-ICP, developed by the Instituto Clodomiro Picado (Costa Rica), includes in its immunisation protocol the venoms of eight viperid species from North Africa and the Middle East (i.e., *Bitis arietans*, *Cerastes cerastes*, *Cerastes gasperettii*, *Daboia mauritanica*, *Daboia palaestinae*, *Echis coloratus*, *Echis pyramidum* and *Macrovipera lebetina obtusa*). This antivenom has recently undergone extensive preclinical evaluation, and demonstrated high efficacy in neutralising the lethal, haemorrhagic, and procoagulant effects of both homologous and heterologous viperid venoms, including *M. l. obtusa*^195^. An equine polyvalent snake antivenom, named HSGM-PAV, is produced by the Republic of Türkiye Ministry of Health, General Directorate of Public Health, using the venoms of *M. l. obtusa*, *Vipera ammodytes montandoni* and *Montivipera xanthina* as immunising agents^196^. A similar antivenom is also produced by Albila Serum Biological Products, a private company based in Türkiye, which includes an equine F(ab') fragment raised against the same venoms (information available from http://www.albila.com/en/product/4/snake-antisera). However, there are no peer-reviewed data published in the literature regarding its efficacy and cross-neutralisation. Furthermore, an ovine-derived experimental monovalent antibody serum against *M. l. obtusa* venom was able to prevent mortality in envenomed mice, although it did not completely mitigate the histopathological effects of the venom^137^. More convincing results were obtained by the antivenom Inoserp™ Europe produced by Inosan Biopharma S. A. (Spain). Currently listed as 'experimental or investigational product' by the WHO^30^, this antivenom has shown to be highly effective against *Macrovipera* venoms in a variety of assays^35,197^. Additional investigational candidates are the human single-chain variable antibody fragments (scFvs) being developed by Institut Pasteur and the Shahid Beheshti University of Medical Sciences in Iran, against the venom of *M. lebetina* and other medically relevant Iranian snakes^198,199^. Small molecule therapeutics also appear to present promising avenues for the development of novel *Macrovipera* snakebite management strategies. For example, Chowdhury et al.^35^ recently evaluated the efficacy of the metalloproteinase inhibitors Marimastat and Prinomastat in neutralising the Factor X-activating effects of *Macrovipera* venoms possibly caused by svMPs. The authors found that Prinomastat exhibited high effectiveness against the venoms of all considered *Macrovipera* taxa (i.e., *M. l. cernovi*, *M. l. obtusa*, *M. l. turanica*, *M. schweizeri*), whereas Marimastat was overall less effective^35^.

It is recommended to administer antivenom as soon as signs of systemic or severe local envenoming arise, although late administration can still prove beneficial. Signs of systemic envenoming requiring antivenom include spontaneous systemic bleeding, incoagulable blood (20-min whole blood clotting test (WBCT20)), and cardiovascular abnormalities (e.g., hypotension, shock). Symptoms of severe local envenoming (e.g., rapidly progressive swelling) and bites to the extremities (e.g., fingers and toes) also warrant antivenom treatment, particularly in the case of necrosis-inducing species like blunt-nosed vipers^181^.

If the species responsible for the bite is identified, monovalent antivenom is the optimal choice. In cases where the species is unknown, polyvalent antivenoms must be utilised, albeit at a higher dosage, to achieve the same specific neutralising effect as monospecific antivenom. Intravenous infusion enables the monitoring of any potential side effects during the infusion process, allowing for its cessation in the event of anaphylaxis. Even if patients exhibit a good clinical response to the initial dose of antivenom, it is recommended to observe them for several days. Continued absorption of venom may lead to recurrent issues (e.g., haematological alterations) after the serum antivenom concentration has diminished. Children should receive the same dose of antivenom as adults^20,186,189^.

While there are no absolute contraindications to the use of antivenom in patients with life-threatening systemic envenoming, all antivenoms carry a risk of potentially dangerous reactions. However, the incidence of anaphylaxis is expected to be lower than that of direct anaphylactoid reactions induced by the venom^200^. Early reactions, occurring 10–60 min after the commencement of intravenous antivenom administration, are mostly anaphylactoid reactions and should be treated following the guidelines for the treatment of standard anaphylaxis. These severe reactions to antivenom may be mitigated by administering epinephrine prior to antivenom treatment in patients with known increased risk^201^. Pyrogenic or late (serum sickness) reactions may arise, with incidence varying based on factors such as antivenom refinement, dose, and route of administration^20,181,186^. Patients with a history of atopic conditions (e.g., asthma) and those who have previously reacted to equine antisera may be at an increased risk. Pyrogenic reactions, which appear 1–2 h post-treatment, result from pyrogen contamination during antivenom manufacture, and can be managed by reducing the patient’s temperature (e.g., using antipyretic drugs other than aspirin). Late reactions (serum sickness type) typically manifest around 7 days after treatment, and can be treated with antihistamines; however, prednisolone may be administered in severe cases^181^.

## Conclusion

By consolidating the vast amount of information available on *Macrovipera* venoms, this review offers a comprehensive overview of their toxinology, pharmaceutical potential and clinical relevance. After defining the zoological, taxonomic, evolutionary, and ecological context of the three species currently recognised—*M. lebetina*, *M. razii* and *M. schweizeri*—we discuss the venom proteomes currently available for this genus. This enabled us to gain a thorough understanding of the general compositional patterns of blunt-nosed viper venoms, and to detect a pronounced focus on *M. lebetina* in the literature. Indeed, we found a remarkably low amount of information concerning the venoms of *M. razii* and *M. schweizeri*, likely due to the recent description of *M. razii* and the perceived lower medical relevance of *M. schweizeri*. Nonetheless, while earlier toxinology studies predominantly focused on a few *M. lebetina* subspecies, recent research has adopted a broader approach, incorporating venoms from more taxa. This shift has proven fruitful, as the more recent characterisations and functional analyses have underscored the pharmacological potential of nearly all *Macrovipera* venoms. Particularly, promising antimicrobial activity and cytotoxic effects against cancer cells have been highlighted.

Drawing from the available information on the biological activity of *Macrovipera* venoms, snakebite case reports, and epidemiological data, we have detailed the pathophysiological effects of envenoming by blunt-nosed vipers. We have also provided guideline-based recommendations for snakebite management, including first aid, early symptom treatment, and antivenom therapy, applicable to cases involving *Macrovipera*. Finally, we outlined the anti-*Macrovipera* antivenoms currently listed by the WHO, including both outdated and substandard products, as well as newer formulations currently in development and testing.

Looking ahead, it appears fundamental to expand the data available on the composition and activities of *Macrovipera* venoms, particularly focusing on under-studied taxa such as *M. razii* and *M. schweizeri*. Furthermore, analysing larger sample sizes through an individual-based approach is crucial, as it enables the investigation of venom variation at different levels (e.g., between and within taxa and populations). The integration of multiple 'omics' technologies and extensive bioactivity profiling in these investigations would be ideal, particularly for the challenge of resolving *Macrovipera* venom profiles down to the primary structure of their components. This would enhance understanding of structure-function relationships, bioactivity, and antivenom efficacy.

To date, venomics studies on blunt-nosed vipers have predominantly relied on proteomics-guided strategies. Supplementing these approaches with genomic and transcriptomic data as taxon-specific databases for protein identification will enable more precise identification and characterisation of the complete *Macrovipera* toxin repertoire. Furthermore, the expansion of genomic resources, whether as well-annotated reference genomes or low-coverage genomes, will contribute to resolving ongoing systematic uncertainties within the genus and promoting taxonomic stability.

Venom chemistry and activity in snakes are closely linked to their ecology, particularly their prey and feeding habits. Thus, to understand venom within its evolutionary framework, it is essential to consider the natural history and ecology of each species. However, this aspect is often overlooked by toxinologists, and many critical details remain undocumented for numerous venomous taxa, including *Macrovipera*. Therefore, the in-depth venomics research outlined above would benefit from a stronger ecological perspective on blunt-nosed vipers. The biogeographic history and current distribution of *Macrovipera* taxa also emerge as key areas of future research to elucidate their evolutionary history, ecological adaptations, and epidemiological significance. Additionally, further in-depth analysis of the properties and biological activities of both *Macrovipera* crude venoms and individual compounds could greatly benefit biodiscovery efforts. Finally, we advocate for the development of toxin-, species-, and region-specific antivenoms, as well as for the improvement of the quality of existing products; for instance, by including venoms from less-studied *Macrovipera* taxa in immunising mixtures.

## Supplementary information

Supplementary Materials

## Data Availability

No datasets were generated or analysed during the current study.
